# Spiritual intelligence, mindfulness, emotional dysregulation, depression relationship with mental well-being among persons with diabetes during COVID-19 pandemic

**DOI:** 10.1007/s40200-021-00927-8

**Published:** 2021-10-31

**Authors:** Wojujutari Kenni Ajele, Teslim Alabi Oladejo, Abimbola A. Akanni, Oyeyemi Bukola Babalola

**Affiliations:** 1 Department of Psychology, Obafemi Awolowo University, Osun State, Ile-Ife, Nigeria; 2 Department of Mental Health, Federal Medical Centre, Lokoja, Nigeria

**Keywords:** Mental well-being, Depression, Emotional dysregulation, Spiritual intelligence, Mindfulness

## Abstract

**Purposes:**

The study examined the mediation moderated effects of spiritual intelligence and mindfulness on the relationship between emotional dysregulation, depression and mental well-being. It also investigated the mediating effects of mindfulness on the relationship between spiritual intelligence and mental well-being in persons with diabetes.

**Methods:**

A cross-sectional survey was carried out among 636 (age 32–74 years; mean = 40.31; SD = 8.40) people living with diabetes who are registered patients and were attending the clinic in Department of Endocrinology, Ondo State Specialist Hospital, Okitipupa and Federal Medical Centre, Lokoja. The data were analysed using Pearson Multiple correlation and mediation moderated model 29 and mediation model 4 of PROCESS macro. The analyses were carried out with PROCESS macro for IBM/SPSS Version 25.0.

**Results:**

Showed significant and positive direct relationship between depression on mental well-being of person with diabetes, β = 0.39, 95 % CI (0.29, 0.48). Results showed mindfulness (β = 0.00, 95 % CI: 0.00, 0.00) and spiritual intelligence (β = -0.01, 0.00, 95 % CI: -0.01, -0.01) significantly moderated the existing direct relationship between depression and mental well-being of persons with diabetes Results showed significant and positive indirect relationship between depression and mental well-being via emotional dysfunctional of persons with diabetes, β = 0.46, 95 % CI (0.44, 0.48). Results showed mindfulness (β = -0.02, 95 %, CI; -0.03, -0.02) and spiritual intelligence (β = -0.00, 95 %, CI: 00.00, -0.01) significant moderated the existing indirect relationship between depression and mental well-being occurred via emotional dysfunctional of persons with diabetes. Results showed significant direct relationship between spiritual intelligence and mental well-being of persons with diabetes, β = -0.12, 95 %, CI: (0.09, 0.16). Results showed mindfulness significantly mediates the existing direct relationship between spiritual intelligence and mental well-being of persons with diabetes, β = -0.11, 95 %, CI: (0.08, 0.15). Results also significant direct relationship between spiritual and mindfulness of persons with diabetes, β = 0. 0.25, 95 % CI: 0.18, 0.31).

**Conclusion:**

Emotional dysregulation play mediating role of the association between depression and mental well-being of persons with diabetes was moderated by spiritual intelligence and mindfulness. Therefore, the study concludes that pay attention spiritual intelligence and mindfulness in management of diabetes will enhance mental well-being of persons with diabetes.

## Introduction

The widespread of novel virus disease that described as Coronaviruses (COVID-19) found alarming and harmed to persons with diabetes. Shi et al. [39] reported that persons with diabetes constitute 9.8 % of 1,561 COVID-19 patients confirmed between the periods of 1 January 2020 to 8 March 2020 in Wuhan, China. Alessi et al. [3] found tall prevalence of emotional distress and depression among persons with diabetes during the COVID-19. Researchers found diabetes as most common comorbidity in COVID-19 patients [46, 10]. A study found diabetes as one of the risk factors of COVID-19 [39]. Hartmann-Boyce et al. [17] reported that persons with diabetes found to be more increasable at of risk of severity of COVID-19 infection.

Mental well-being is a key psychological component that can enable persons with diabetes to potentially cope with the normal stresses of life, diabetes-related distress, physical, emotional and psychological distress. Mental well-being in persons with diabetes can be describes as internal and external factor that can be influence by various psychological factors such depression, emotional dysfunctions, spiritual intelligence and mindfulness. Depression is a psychological condition that been found to be common with persons with diabetes; if not well managed that may capable of ruin patients emotional, economical, spiritual, physical and social well-being.

Diabetes increasing the risk for depression, diabetes also can make depressive symptoms worse [31]. Depression co-occurrence with diabetes associated with poor mental well-being and premature death; is found as a main health challenge for persons with diabetes [47]. COVID-19 pandemic may add to the burden being faced by people with diabetes Depression is a mental disorder that individual experience symptoms of depressed or sad mood, diminished interest in activities that used to be pleasurable, weight gain or loss, psychomotor agitation or retardation, fatigue, inappropriate guilt, difficulties concentrating, as well as recurrent thoughts of death that occur most of the day, nearly every day, for at least two weeks [5].

According to the World Health Organisation [48] approximately 382 million people have diabetes mellitus and 350 million among them were suffering from depression worldwide. Depression is mental illness found to be common in persons with diabetes, the majority of the patients are untreated and underdiagnosed [8, 21] may weakened and detrimental to their mental well-being.

Recent growing body of literature reported the high prevalence of depression in persons with diabetes [12, 33, 42, 38]. However, the relationship between depression and mental well-being in persons with diabetes has not extensively examined. Smith et al. [40] finding suggested that depression and anxiety main contributors of poor mental well-being of UK residents during the period of COVID-19 outbreak.

The co-occurrence between diabetes and depression can be bidirectional because each can put a patient at risk for the other. Some of the symptoms of depression, such as weight gain, overeating and decreased interest in self-care, can worsen the symptoms of diabetes [31]. People with diabetes and depression have more severe diabetes symptoms than people who have diabetes alone [31]. Poor glycaemic control and problematic self-care behaviour found to be associated depressive symptoms in persons with diabetes [15]. The fatigue of managing diabetes can also lead to depression that adversely affects the mental well-being of persons with diabetes [31]. Meanwhile, studies have not determined the exacerbating role by emotional dysregulation of bidirectional relationship between the depression and mental well-being of persons with diabetes.

Persons with diabetes inability to control negative emotions or emotional dysregulation can associate with excessive worry and concerns about potential emergence of diabetes related complications, erratic blood glucose, hypoglycaemic episodes and feelings of depressive symptoms [23, 22]. Persons with diabetes may experience emotional dysregulation when patients failed to control or regulate their emotional responses to diabetes-related psychological distress. Persons with diabetes also suffers emotional dysregulation may experience uncontrollable negative emotions by overreacting to diabetes related self-care challenges such as bursts of anger, crying, accusing, feeling frustrated, hopeless, angry, guilty, fearful and passive-aggressive behaviours.

Emotion dysregulation is an uncontrolled or managed fervent negative emotional state, which may risk persons with diabetes to develop of depressive symptoms and poor mental well-being [2, 6]. Powers et al. [35] findings revealed significant relationship between emotion dysregulation, higher C-reactive protein (CRP), body mass index, trauma exposure, and major depressive disorder (MDD) diagnosis among women with diabetes. Young-Hyman et al. [50] found significant relationship between depressive symptoms, emotion dysregulation and bulimic symptoms in youth with diabetes. Yilmaz Kafali et al. [49] found significant relationship emotional dysregulation and psychiatric comorbidities associated with person with type 1 diabetes mellitus (T1DM). Abravanel and Sinha [1] found significant mediating role of emotion dysregulation on the relationship between lifetime cumulative adversity and depressive symptomatology.

American Psychological Association [4] described mindfulness as a state of mind where persons with diabetes consciously direct attention to diabetes-related self-care by accepting and non-judgmental of their current health status. Mindfulness has been found significant effective in reducing alcohol and cannabis consumption through mindfulness-meditation interventions [13]. Pearson et al. [34] findings showed that individual with higher mindfulness tendency experience lower levels of anxiety, depression, and alcohol-related consequences than individual with low mindfulness tendency.

Kian et al. [25] finding showed that mindfulness significant associated with mild level of depression, anxiety, Fasting Blood Sugar (FBS), Hemoglobin A1C (HbA1C) and enhanced emotional well-being and glycemic control through mindfulness-based stress reduction (MBSR) in persons with diabetes. Similarly, a meta-analysis conducted by Guo et al. [16] findings showed that mindfulness-based interventions have significant effect of reduce diabetes distress in adults with diabetes. Hsieh et al. [19] findings showed that mindfulness has a significant moderating effect on the relationship between caregiving stress and depressive symptoms in caregivers of patients with lung cancer. Lee and Zelman [28] findings showed that mindfulness has a significant moderating effect on the boredom proneness of depression, anxiety and stress. Lu et al. [29] findings showed that mindfulness significantly moderate the perceived stress on emotional exhaustion, depression, anxiety among Chinese intensive care nurses. Zhong et al. [51] found moderating effect of mindfulness on the existing relationship between perceived stress and psychological symptoms in Chinese digestive tract cancer patients.

Recent studies also found effect of spiritual intelligence enhancing individual physical and emotional health outcomes [30, 24]. Diabetes-related spiritual intelligence can be described as persons with diabetes effectively exploited their spiritual abilities and resources with the aim at boosting diabetes-related distress management and diabetes-related complications coping skills and improve the performance of daily self-management and mental well-being [20, 36]. Rahmanian et al. [37] findings showed that spiritual intelligence has significant positive impact on self-management in adolescents with diabetes.

Cisheng et al. [11] findings showed that spiritual intelligence has significant moderating effect on the association between emotional intelligence and identity development in adolescent students. Chaleshtari et al. [9] showed that spiritual intelligence has a significant effect of improving self-efficacy and social responsibility in students through group training. Sogolitappeh et al. [41] findings showed that spiritual intelligence significant relationship between emotional intelligence and resilience. These existing studies.

## Objectives of the study

The main aim of the study was to assess the roles of some risk factors associated with mental well-being of persons with diabetes during covid-19 pandemic. This is because the pandemic came with worries and anxiety that can play significant role in the development and exacerbation of severe diabetes complications. People with diabetes may have COVID-19-specific worries related to their diabetes which could be associated with several risk factors. Thus, the specific objectives of this study were to examine the relationship between spiritual intelligence, mindfulness, emotional dysregulation, depression and mental well-being; examine the mediation-moderated role of spiritual intelligence and mindfulness on the relationship between emotional dysregulation, depression and mental well-being and investigate mediating role of mindfulness on the relationship between spiritual intelligence and mental well-being in persons with diabetes.

## Methods

### Design

The study conducted a cross-sectional survey design. Primary data was collected through the administration of a set of standardised psychological scales on a convenient sample of the study population. Also, patients’ data such as diagnosis, and socio-demographic were collected from their medical files. Each file contains patient’s age, sex, type of diabetes, type of management.

### Participants

A total of 636 diabetes patients receiving follow-up clinical treatment in Endocrine unit, Ondo State Teaching Hospital, Akure and Federal Medical Centre, Lokoja were purposively selected for the study. Ondo State Teaching Hospital, Akure and Federal Medical Centre, Lokoja provide wide range of medical services to patients in different departments. A total of 380 (382 male and 254 female) participants were selected for the study using the convenient sampling technique whereby only patients available on four consecutive clinic days were approached individually and those who consented were included in the study.

The sample comprised of 343(53.9 %) persons with T2DM and 293(46.1 %) and person with T1DM participants in study (presented in Table 1). The majority 255(40.1 %) of the respondents were receiving Insulin injection type of management, 221(34.7 %) of the respondents were receiving oral drug type of management while the remaining 106(25.2 %) of the respondents were receiving Insulin injection and oral drug type of management (presented in Table 1). The educational level of majority 217(34.1 %) of the respondents were National College of Education (NCE) and Ordinary National Diploma (OND), 194(30.5 %) of the respondents were secondary school, 110(17.3 %) of the respondents were higher degree, 73(11.5 %) of the respondents were modern school holders and while the remaining 42(6.6 %) of the respondents were primary. The age of the 636 respondents ranged from 32 to 74 years with the mean = 38.6 and SD = 6.07.

### Inclusion and exclusion criteria

Eligible participants included in study were outpatients who had been confirmed the diagnosis of either T1DM or T2DM, who was receiving follow-up clinical treatment regularly in the Endocrine unit, Ondo State Teaching Hospital, Akure and Federal Medical Centre, Lokoja. Participants that were excluded from the study are those who cannot read and write English Language, persons with gestational diabetes and individual with serious health condition and complication of diabetes such as diabetic neuropathy, diabetes eye disease, diabetes nephropathy, and cardiovascular diseases and individual who are not regular visitor of diabetic clinic in the selected hospitals.

**Table 1:** Summary of analysis of respondents clinical characteristics (*N* = 636)

Variables	Groups	Frequency	Percentages (%)
Types of Diagnosis	Type 1 Diabetes	293	46.1
	Type 2 Diabetes	343	53.9
Medical Management of Diabetes	Insulin	255	40.1
	Oral	221	34.7
	Both Insulin and Oral	160	25.2

### Instruments

#### Short Warwick-Edinburgh Mental Well-Being Scale (SWEMWBS-7)

the Short Warwick-Edinburg Mental Well-Being Scale (WEMWBS) developed by Tennant et al. [43] was uses to measure the study participant’s mental well-Being. The SWEMWBS consists of 7-item that measure individual subjective well-being and psychological functioning. These 7-items are worded positively and address aspects of positive mental health. Each item is rated on a 5-point Likert-type scale ranging from 1 (None of the time) to 5 (All of the time).the SWEMWBS 7-items scored by summing responses to each item. The scores range from 7 minimum to 35 maximum and higher scores indicates higher levels of mental well-being. The SWEMWBS demonstrated good internal consistency with Cronbach’s alpha coefficient = 0.89 [44].

#### Patient Health Questionnaire (PHQ-9)

Patient Health Questionnaire is a self-report 9-items measure that incorporated DSM-IV depression diagnostic criteria by Kroenke et al. [27] was used to assess participant’s depression. The responses to each item are rated on a 4-point Likert-type scale; (0 = not at all, 1= several days, 2 = more than half the days, 3 = nearly every day). The PHQ-9 can be administered to groups or individuals within 20 min. Scores of 0-4 indicate ‘‘minimal’’ depression, 5–9 indicate “mild” depression, 10-14 indicate “mild” depression, 15-19 indicates “moderate” depression, and 20–27 indicate “severe” depression [27]. Khamseh et al. (2011) found high internal consistency for PHQ-9-Cronbach’s Alpha = 0.86.

#### Difficulties in Emotion Regulation Scale (ERS-18)

The brief version of the Difficulties in Emotion Regulation Scale (DERS-18) developed by Victor and Klonsky [45] was used to measure participants emotional dysregulation. The brief version of the Difficulties in Emotion Regulation Scale (DERS-18) is 18-items self-report developed to emotional dysregulation in the general population. Each item is rated on a 5-point Likert-type scale; (1= almost never (0-10 %), 2= sometimes (11-35 %), 3 = about half the time (36-65 %), 4 = most of the time (66-90 %), 5 = almost always (91-100 %) and higher scores indicate more difficulty in emotion regulation. The DERS-18 has constantly demonstrated good internal consistency with Cronbach’s that alpha ranging between 0.69 and 0.80 [45] across samples.

#### Spiritual Intelligence Inventory (SISI)

The Spiritual Intelligence Self Report Inventory (SISI) developed by King [26] was used to assess participant’s spiritual intelligence. The SISI comprised 24-items which each item is rated on a 5-point Likert scale: 0=not at all true to me, 1= not very true of me, 2= somewhat true of me, 3= very true of me and 4 = completely true of me. The SISI total scores range from 0 to 96 and higher scores indicate high spiritual intelligence. The SISI has good internal consistency Cronbach’s alpha of 0.95 [26]. Nurayunee et al. [32] found good internal consistency with Cronbach’s that alpha ranging between 0.60 and 0.80.

#### Mindful attention awareness scale (MAAS)

The Mindful Attention Awareness Scale (MAAS) was developed by Brown and Ryan [7] was used to measure participant’s mindfulness. The MAAS is a 15-item scale developed to measure a core characteristic of dispositional mindfulness. Each item is rated on a 6-point Likert-type scale; 1= almost always, 2= very frequently, 3 = somewhat frequently, 4 = somewhat infrequently, 5 = very infrequently, 6 = almost never. The MAAS can be administered within 10 min or less to complete. The MAAS total scores range from 15 to 90 which higher scores indicate high mindfulness. The MAAS has good internal consistency alpha coefficient of 0.82 [7].

### Procedure

Two ethical clearances were obtained from the Research Ethics Committee of the two selected hospitals. Participants gave informed consent before they were enrolled in the study. The researchers were introduced by the consultant to the nurses, patients and other members of the staff in the department of endocrinology. This was to ensure the maximum support of the nurses and other staff of the hospital in enlisting patients who meet the criteria for inclusion in the study. The study questionnaire was administered individually by the researchers on clinic days (Thursdays) within the hospital premises.

The participants filled questionnaires during their waiting time to see their doctors for consultations. Although waiting times were often long, some of the participants completed their questionnaires after consultations. In order to ensure an anonymous process while collecting the data, a drop box was placed in the waiting room. Participants were informed about the drop box and the importance of anonymity. The researchers retrieved the questionnaires from the participants who did not used the drop box and also packed those ones that were dropped in the drop box at the end of each clinic day. A researcher was always present in the waiting room during the data collection process, in order to give clarifications and also provide answers to all the participants’ questions about the study. Out of 656 questionnaires administered, 6 were not retrieved, 14 were discarded due to errors from the participants and 636 were used for the final analysis. The study data were collected between 19th of March to 28th of May, 2020.

### Data analysis

The data were analysed using Pearson Multiple correlation and mediation moderated model 29 and mediation model 4 of PROCESS macro. The analyses were carried out with ROCESS macro for IBM/SPSS Version 25.0.

## Results

Table 2 presented the results of study hypothesis that states there is no relationship between spiritual intelligence, mindfulness, emotional dysregulation, depression and mental well-being among persons with diabetes. The results showed mental well-being positive significantly correlated with spiritual intelligence (r = 0.34, p < 0.05), mindfulness (r = 0.31, p < 0.05), emotional dysregulation (r = 0.90, p < 0.05) and depression (r = 0.26, p < 0.05). The results showed depression positive significantly correlated with spiritual intelligence (r = 0.45, p < 0.05), mindfulness (r = 0.56, p < 0.05) and emotional dysregulation (r = 0.16, p < 0.05). The results also showed emotional dysregulation positive significantly correlated with spiritual intelligence (r = 0.37, p < 0.05) and mindfulness (r = 0.21, p < 0.05). The results further showed positive significant correlation between mindfulness and spiritual intelligence (r = 0.28, p < 0.05). The alternative hypothesis which states there is significant relationship between spiritual intelligence, mindfulness, emotional dysregulation, depression and mental well-being among persons with diabetes was accepted, while the null hypothesis was rejected.

**Table 2:** Summary of multiple Pearson correlation analysis between the Spiritual intelligence mindfulness, emotional dysregulation, depression and mental well-being (*N =* 636)

Variables	Mean (SD)	(1)	(2)	(3)	(4)
SI (1)	20.58 (20.19)				
Mindfulness (2)	31.45 (18.07)	0.28^**^			
EDRS (3)	23.14 (18.83)	0.37^**^	0.21^**^		
Depression (4)	8.42 (8.39)	0.45^**^	0.56^**^	0.16^**^	
MWB	20.94 (8.85)	0.34^**^	0.31^**^	0.90^**^	0.26^**^

Note: Spiritual intelligence (SI), emotional dysregulation (EDRS) and mental well-being (MWB)

Table 3 presented results of study hypothesis that states spiritual intelligence and mindfulness have no significant moderating role on indirect relationship between depression and mental well-being occur through emotional dysregulation among persons with diabetes during covid-19 pandemic. there is no significant mediating role of mindfulness on the relationship between spiritual intelligence and mental well-being in persons with diabetes.

**Fig. 1:**
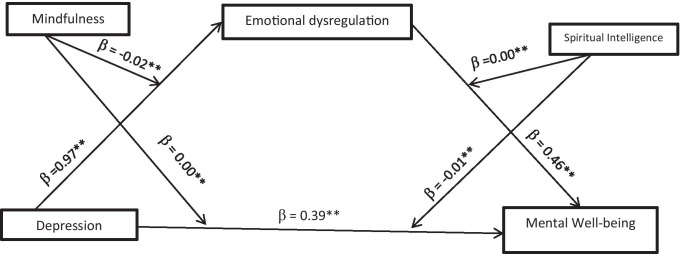
Theoretical research model with standard coefficients (β) Model 29 [18]

This hypothesis was tested with Model 29 mediation, moderation, and conditional process analysis [18]. Emotional dysregulation among persons with diabetes was explained by the model at a rate of 10 % (R^2^ = 0.10) of the total variance of depression, *F* (3, 632) = 22.38, p < 0.05. The showed significant positive direct relationship between depression and emotional dysregulation (β = 0.97, p < 0.05), [95 % Cl: = 0.62 (1.32)]. The results also showed that the existing direct relationship between depression and emotional dysregulation was significant and negatively moderated by mindfulness (β = -0.02, p < 0.05), [95 % Cl: = -0.03 (-0.02)] see Fig. 1. The result showed significant and positive direct effects of depression on mental well-being (β = 0.39, p < 0.05), [95 % Cl: = 0.29(0.48)] which was significantly moderated by mindfulness (β = 0.00, p < 0.05), [95 % Cl: = 0.00 (0.00)] and spiritual intelligence (β = -0.01, p < 0.05), [95 % Cl: = -0.01 (-0.01)].

Table 3 results also showed that mental well-being among persons with diabetes was explained by the model at a rate of 87 % (R^2^ = 0. 87) of the total variance of depression, *F* (7, 628) = 599.05, p < 0.05. The results showed significant indirect relationship between of depression and mental well-being via emotional dysregulation, (β = 0.46, p < 0.05), [95 % Cl: = 0.44 (0.48)] which was significant and positively moderated by spiritual intelligence (β = 0.00, p < 0.05), [95 % Cl: = 0.00 (0.01)] see Fig. 1. The alternative hypothesis which states spiritual intelligence and mindfulness have significant moderating role on indirect relationship between depression and mental well-being occur through emotional dysregulation among persons with diabetes during covid-19 pandemic was accepted, while the null hypothesis was rejected.

**Table 3:** Summary of moderated mediation analysis of mindfulness, SI, Depression and EDRS predicting mental well-being (model 29 of PROCESS macro; *N =* 636)

	Explained Variables					
	EDRS				Mental Well-being	
Model	Β	*SE*	95% CI	β	*SE*	95% CI
			LLCI (ULCI)			LLCI (ULCI)
Constant	7.09**	2.11	2.94 (11.24)	7.55**	0.39	6.78 (8.32)
Depression	0.97**	0.18	0.62 (1.32)	0.39**	0.05	0.29(0.48)
EDRS				0.46**	0.01	0.44 (0.48)
Mindfulness	0.52**	0.07	0.38 (0.67)	0.05**	0.02	0.02 (0.08)
Depression × Mindfulness	-0.02**	0.00	-0.03 (-0.02)	0.00*	0.00	0.00 (0.00)
SI				0.15**	0.02	0.12 (0.19)
Depression × SI				-0.01**	0.00	-0.01 (-0.01)
EDRS × SI				0.00**	0.00	0.00 (0.01)
	R^2^	0.10	R^2^		0.87	
	*F(df)*	*F*(3, 632) = 22.38	*F(df)*		*F*(7, 628) = 599.05**	

Note: Spiritual intelligence (SI), emotional dysregulation (EDRS) and mental well-being (MWB)

Table 4 presented the results showed index of moderated mediation was significant [95 % Cl: = 0.00 (0.00)], indicating that the indirect effect of depression on mental well-being in persons with diabetes through EDRS was moderated by mindfulness and spiritual intelligence. Table 4 also presented the conditional indirect effect on values of the moderators were calculated based on one standard deviation above (+SD) and one standard deviation below (-SD). The results revealed significant indirect effect of levels of mindfulness; low mindfulness (β = 0.60, p < 0.05), [95 % Cl: = 034 (0.86)], moderate mindfulness (β = 0.19, p < 0.05), [95 % Cl: = -0.01(0.40)], and high mindfulness (β = 0.12, p < 0.05), [95 % Cl: = -0.49 (-0.01)]. The result also showed significant indirect effect of levels of mindfulness; low spiritual intelligence (β = 0.46, p < 0.05), [95 % Cl: = 0.44 (0.48)], moderate spiritual intelligence (β = 0.39, p < 0.05), [95 % Cl: = 0.37 (0.40)], and high spiritual intelligence (β = 0.31, p < 0.05), [95 % Cl: = 0.28(0.34)]. Thus, the indirect effect of depression on mental well-being in persons with diabetes through EDRS and the direct effect moderation can be achieved through low, moderate and high levels of mindfulness and spiritual intelligence in persons with diabetes.

**Table 4:** Summary of analysis of conditional indirect effect and Index of moderated mediation

Explained Variables						
Levels	Mindfulness			Spiritual Intelligence		
Model	Β	*SE*	95 % CI	β	*SE*	95 % CI
			LLCI (ULCI)			LLCI (ULCI)
Low (−1SD)	0.60**	0.13	034 (0.86)	0.46**	0.01	0.44 (0.48)
Moderate	0.19**	0.10	-0.01(0.40)	0.39**	0.01	0.37 (0.40)
High (+1SD)	-0.25**	0.12	-0.49 (-0.01)	0.31**	0.01	0.28(0.34)

**Fig. 2:**
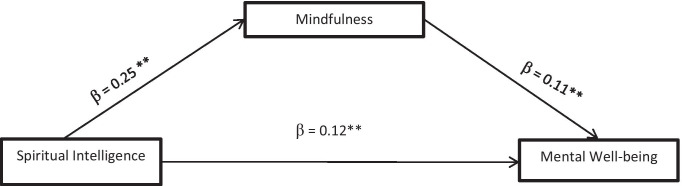
Theoretical research model with standard coefficients (β) Model 4 [18]

Table 5 presented the results of the hypothesis that states there is no significant mediating role of mindfulness on the relationship between spiritual intelligence and mental well-being in persons with diabetes. This hypothesis was tested with Model 4 mediation, moderation, and conditional process analysis [18]. Mental well-being among persons with diabetes was explained by the model at a rate of 8 % (R^2^ = 0.08) of the total variance of mindfulness, *F* (2, 633) = 63.66, p < 0.05. Mediation analysis presented in Table 5 revealed significant positive direct relationship between spiritual intelligence and mindfulness (β = 0.25, p < 0.05), [95 % Cl: = 0.18 (0.31)] see Fig. 2. The overall model of the direct relationship accounted 08 % of the total variance of mindfulness, *F* (1, 634) = 51.68, p < 0.05. The results also showed significant positive direct relationship between spiritual intelligence and mental well-being (β = 0.12, p < 0.05), [95 % Cl: = 0.09 (0.16)] and further revealed significant and positive direct relationship between mindfulness and mental well-being (β = 0.11, p < 0.05), [95 % Cl: = 0.08 (0.15)] see Fig. 2. This implies that mindfulness can strengthen the relationship between spiritual intelligence and mental well-being in persons with diabetes.

**Table 5:** Summary of mediation analysis of mindfulness and SI on mental well-being (model 4 of PROCESS macro; *N =* 636)

Explained Variables						
	Mindfulness			Mental Well-being		
Model	β	*SE*	95 % CI	Β	*SE*	95 % CI
			LLCI (ULCI)			LLCI (ULCI)
Constant	26.39**	0.99	24.46 (28.33)	14.85**	0.67	13.54 (16.17)
SI	0.25**	0.03	0.18 (0.31)	0.12**	0.02	0.09 (0.16)
Mindfulness				0.11**	0.02	0.08 (0.15)
	R^2^	0.08		R^2^	0.17	
	* F(df)*	*F*(1, 634) = 51.68**		*F(df)*	*F*(2, 633) = 63.66**	

Note: SI = spiritual intelligence

## Discussion

The aim of the study was to examine the mediating and moderating effects of spiritual intelligence and mindfulness on the relationship between emotional dysregulation, depression and mental well-being as well as investigated mediating effects of mindfulness on relationship between spiritual intelligence and mental well-being in persons with diabetes during COVID-19 Pandemic in Nigeria.

The study results revealed the significant mediation moderated effects of spiritual intelligence and mindfulness on the relationship between emotional dysregulation, depression and mental well-being which is consistence with the previous findings of Lu et al. [29] who find that mindfulness has significant moderation of perceived stress on emotional exhaustion, depression, anxiety among Chinese intensive care nurses. Also, finding is in congruent with Hsieh et al. [19] findings showed that mindfulness has significant moderating effects on the relationship between caregiving stress and depressive symptoms in caregivers of patients with lung cancer. finding is in congruent with Yilmaz Kafali et al. [49] findings showed that significant relationship emotional dysregulation and psychiatric comorbidities associated with person with type 1 diabetes mellitus (T1DM). The result is in congruent with Abravanel and Sinha [1] findings showed that significant mediating role of emotion dysregulation on the relationship between lifetime cumulative adversity and depressive symptomatology.

The study results revealed the significant and positive mediating effects of mindfulness on relationship between spiritual intelligence and mental well-being in persons with diabetes. The study results supported findings that showed significant mediating effect of emotion dysregulation on relationship between attention-deficit/hyperactivity disorder (ADHD) symptoms and emotional impulsivity. it also in consistence with Adrian et al. (2010) findings that showed emotional dysregulation as risk factor for adolescents to develop non-suicidal self-injury (NSSI) and mediating the influence of interpersonal problems through the family and peer relational problems.

## Conclusions

Emotional dysregulation play mediating role of the association between depression and mental well-being of persons with diabetes was moderated by spiritual intelligence and mindfulness. Therefore, the study concludes that pay attention spiritual intelligence and mindfulness in management of diabetes will enhance mental well-being of persons with diabetes.
